# The Effects of Long-Term Saturated Fat Enriched Diets on the Brain Lipidome

**DOI:** 10.1371/journal.pone.0166964

**Published:** 2016-12-01

**Authors:** Corey Giles, Ryusuke Takechi, Natalie A. Mellett, Peter J. Meikle, Satvinder Dhaliwal, John C. Mamo

**Affiliations:** 1 Curtin Health Innovation Research Institute, Curtin University, Perth, Western Australia, Australia; 2 School of Public Health, Faculty of Health Sciences, Curtin University, Perth, Western Australia, Australia; 3 Metabolomics Laboratory, Baker IDI Heart and Diabetes Institute, Melbourne, Victoria, Australia; Universitat de Lleida-IRBLLEIDA, SPAIN

## Abstract

The brain is highly enriched in lipids, where they influence neurotransmission, synaptic plasticity and inflammation. Non-pathological modulation of the brain lipidome has not been previously reported and few studies have investigated the interplay between plasma lipid homeostasis relative to cerebral lipids. This study explored whether changes in plasma lipids induced by chronic consumption of a well-tolerated diet enriched in saturated fatty acids (SFA) was associated with parallel changes in cerebral lipid homeostasis. Male C57Bl/6 mice were fed regular chow or the SFA diet for six months. Plasma, hippocampus (HPF) and cerebral cortex (CTX) lipids were analysed by LC-ESI-MS/MS. A total of 348 lipid species were determined, comprising 25 lipid classes. The general abundance of HPF and CTX lipids was comparable in SFA fed mice versus controls, despite substantial differences in plasma lipid-class abundance. However, significant differences in 50 specific lipid species were identified as a consequence of SFA treatment, restricted to phosphatidylcholine (PC), phosphatidylethanolamine (PE), alkyl-PC, alkenyl-PC, alkyl-PE, alkenyl-PE, cholesterol ester (CE), diacylglycerol (DG), phosphatidylinositol (PI) and phosphatidylserine (PS) classes. Partial least squares regression of the HPF/CTX lipidome versus plasma lipidome revealed the plasma lipidome could account for a substantial proportion of variation. The findings demonstrate that cerebral abundance of specific lipid species is strongly associated with plasma lipid homeostasis.

## Introduction

The brain is highly enriched in lipids, supporting structural, biochemical and cell signalling functions [1]. Bioactive lipids within the brain are shown to be pivotal for central nervous system homeostasis by modulating neurotransmission, synaptic plasticity, enzyme function, ion channel activities, gene expression and inflammation [2–4]. Changes in cerebral lipid homeostasis are widely reported to be associated with neurodegenerative disorders, and several studies also suggest significant changes in brain lipids with non-pathological ageing [5–10].

The regulation of the cerebral lipidome is poorly understood. Some studies suggest that the isolated organ status of brain limits substantial shifts in brain lipid homeostasis. However, longer-term feeding studies (8–12 weeks duration) in animal models with fat formulated diets suggest a cerebral response within the brain lipidome [11–14]. With severe dietary n-3 fatty acid deficiency, several laboratories reported changes in the phospholipid composition of the brain [15–18]. In other studies, Rabiei et al. (2013) reported in a rodent model of stroke, that pre-treatment with dietary virgin olive oil influenced the brain lipidome in a dose dependent manner [19]. Such observations demonstrate that the brain lipidome is at least partially modulated as a consequence of changes in peripheral or dietary lipids [11, 20, 21].

The functional properties of brain capillaries ordinarily strictly regulate kinetics of plasma macromolecules such as lipoproteins between blood and brain [22]. Characterized by tightly apposed junctional proteins between adjacent capillary endothelial cells, non-specific intercellular kinetics across the capillary plasma membrane is normally restricted. However, the brain can rapidly take up free fatty acids from circulation and cerebral endothelial cells express lipases which can hydrolyse lipoprotein associated lipids for uptake. Other potential blood-to-brain lipid kinetic pathway includes transcytotic vesicles forming on the plasma membrane of capillary endothelia with extrusion occurring on the subluminal basolateral membrane [23]. Moreover, attenuated expression of endothelial junctional proteins may result in greater transendothelial rates of molecular transport. This phenomenon has been described in neurological disorders [24] and also in brain capillaries of aged rodent models [25, 26]. The latter was reported to be exacerbated by high fat feeding with diets enriched in saturated fats or cholesterol [27].

Chronic ingestion of Western styled diets enriched in saturated fats are causally associated with a range of neurodegenerative disorders including vascular dementia and Alzheimer’s disease [28, 29]. The mechanisms underpinning this association are not completely understood but broadly include capillary dysfunction; neurovascular inflammation; altered redox state and heightened oxidative stress [30–33]. With chronic ingestion of pro-inflammatory fat enriched diets, it is a reasonable proposition to suggest that changes of the brain lipidome may be realized and causally associated with some of the indicated mechanistic pathways. Cerebral sequelae that may reflect changes in the brain lipidome following long-term consumption of high-fat diets may be broad and include compromised insulin signalling [21, 34], neuronal apoptosis [12], and poorer cognitive performance [35, 36].

The rapid development of lipidomics provides an unprecedented opportunity to consider the brain lipidome in the context of central nervous system function. Lipidomic studies show marked differences in brain lipid species in several neurodegenerative disorders [7, 8, 37–39], however it is unclear whether these changes are causally associated with the onset or progression of said disorders. The hippocampal formation (HPF) is central to episodic and associative memory and significantly compromised in cognitively deficient subjects [40]. The cerebral cortex contribute to executive functioning and semantic functioning [41]. To consider the putative changes within the brain lipidome in the physiological context of dietary behaviour, this study comprehensively explores cerebral lipid homeostasis in otherwise healthy wild-type mice maintained on ordinary chow, or a diet enriched with saturated fat (SFA) for six months. In addition, we consider the potential of the plasma lipidome to explain the variance observed in the HPF and CTX lipidomes.

## Methods

### Animals

All experimental procedures were approved by the Curtin University Animal Ethics Committee and conducted in accordance with National Health and Medical Research Council (NHMRC) guidelines. Twenty C57BL/6 mice where obtained from the Animal Resource Centre (ARC; Murdoch). They were housed in an accredited animal holding facility with 12 hour light/dark cycles, at ambient temperature of 22 degrees Celsius. Mice had ad libitum access to water and their respective diets. At 8 weeks of age, mice were randomly assigned to one of two groups, receiving either AIN-93M control diet (9% energy from canola oil), or a modified AIN-93M chow containing 40% energy from cocoa butter (Glen Forrest Stock Feeds). Table 1 indicates the approximate fatty acid composition of the diets and quantity of fatty acids consumed per kilogram of diet.

**Table 1:** Lipid composition of regular chow and saturated fat enriched chow.

	Fatty acid composition (%)		Quantity per kg (g/kg)	
	Control diet	High fat diet	Control diet	High fat diet
C12 or less	0.2	4.7	0.1	9.6
C14:0	0.2	0.2	0.1	0.5
C16:0	4.4	24.4	1.7	49.7
C18:0	2.1	34.5	0.8	70.5
C20:0	0.5	1.1	0.2	2.3
C16:1	0.1	0.2	0.04	0.5
C18:1	60.6	31.3	24	64
C20:1	1.0	0.0	0.4	0
C18:2 n6	20	3.2	7.9	6.5
C18:3 n3	9.5	0.2	3.8	0.5
SFA	7.1	63.7	2.8	129.9
MUFA	62.3	32.8	24.9	66.9
PUFA	30.6	3.5	12.3	7.2

Approximate fatty acid composition of regular AIN-93M chow and saturated fat enriched chow used in this study. Percentage of fatty acid composition is relative to total fat content (4% w/w for regular chow and 20% w/w for SFA enriched chow). Accounting for difference in total fat content, the quantity of fatty acids in grams per kilogram of diet is shown.

### Sample isolation and preparation

Mice were maintained on their respective diets for six months. One mouse was removed from the study prior to completion due to health issues unrelated to the study design. Mice were administered an intraperitoneal dose of pentobarbital and following complete anaesthesia, blood was collected through cardiac puncture into EDTA containing tubes. Plasma was separated through centrifugation and frozen at -80 degrees Celsius for further analysis. Following exsanguination, brains were rapidly excised, washed in ice-cold PBS and left hemispheres snap frozen in liquid nitrogen. Using a commercial brain block, the left frozen hemispheres were sectioned into 1 mm coronal slices. Under a stereotaxic microscope, sections of the S2 cerebral cortex and hippocampus were isolated and weights recorded. Isolated regions were diluted in 10 volumes of ice cold phosphate buffered saline (10 mM phosphate, 137 mM NaCl, 2.7 mM KCl; pH 7.4), homogenized and frozen for further analysis.

### Extraction of lipids

The extraction of lipids was performed by a signle phase chloroform:methanol extraction as described previously [42]. In brief, 10 uL of plasma or 10 uL of brain homogenate (containing approximately 20 ug of protein) was transferred to an eppendorf tube with 10 uL of internal standard mixture [42]. The internal standard mix included lipid species from the lipid classes dihydroceramide (dhCer d18:0/8:0), ceramide (Cer d18:1/17:0), monohexosylceramide (MHC d18:1/16:0d3), dihexosylceramide (DHC d18:1/16:0d3), trihexosylceramide (THC d18:1/17:0), sphingomyelin (SM d18:1/12:0), phosphatidylcholine (PC 13:0/13:0), lysophosphatidylcholine (LPC 13:0), phosphatidylethanolamine (PE 17:0/17:0), lysophosphatidylethanolamine (LPE 14:0), phosphatidylserine (PS 17:0/17:0), phosphatidylglycerol (PG 17:0/17:0), cholesterol ester (CE 18:0d6), cholesterol (COHd7), diacylglycerol (DG 15:0/15:0), triacylglycerol (TG 17:0/17:0/17:0) and bis(monoacylglycero)phosphate (BMP 14:0/14:0). Chloroform/methanol (2:1; 20 volumes) was added to each sample, followed by rotary mixing (10 minutes), sonication (30 minutes) and allowed to stand (20 minutes) at room temperature. Samples were centrifuged (16,000 x g, 10 minutes) and the supernatant collected and dried under nitrogen gas at 40°C. Samples were reconstituted with 50 uL of water saturated butanol and sonicated (10 minutes), followed by 50 uL methanol (with 10 mM ammonium formate). Extracts were centrifuged (3350 x g, 5 minutes) and the supernatant transferred to 0.2 mL glass vials with Teflon caps ready for analysis.

### Mass spectrometric analysis of lipids

Analysis of lipids were conducted using liquid chromatography electrospray ionisation-tandem mass spectrometry on an Agilent 1200 UHPLC coupled to an AB Sciex Q/TRAP 4000 mass spectrometer with a turbo-ionspray source as comprehensively described previously [42, 43]. Liquid chromatographic separation was performed on a Zorbax C18 column (1.8 μm, 50x2.1 mm; Agilent Technologies). Mobile phase solutions consisted of tetrahydrofuran:methanol:water (A, 30:20:50; B, 75:20:5) containing 10 mM ammonium formate. Solvent flow was set at 300 μL/minute using the gradient: 0% B to 100% B in 8 minutes, a further 2.5 minutes at 100%, a return to 0% B over 0.5 minutes and a 3 minute hold at 0% B prior to the next injection. Diacylglycerols and triacylglycerols were separated by an isocratic flow at 85% B over 6 minutes. Briefly, detection of lipids was performed with scheduled multiple reaction monitoring (MRM) in positive ion mode using Analyst 1.5 (AB Sciex) (details in S1 Table). Precursor-product ion pairs (identified from precursor and neutral loss scans, as described previously [42]) were continuously scanned over their elution period with a 30 second window. The concentration of individual lipid species was determined by relating the integrated peak areas to the appropriate internal standards in MultiQuant 2.1 (AB Sciex). Quality control samples were included every 20 samples to assess repeatability and drift. In total, 348 lipids where detected and quantitated from each plasma, cerebral cortex and hippocampal sample.

### Data analysis

Individual lipid specie concentrations were expressed as picomoles per mg wet weight for brain samples and picomoles per microliter for plasma samples. Total lipid concentration of each class was calculated by summing across individual species in that class. Pre-treatment of data included removal of lipids species below the limit of detection from the dataset. Univariate comparisons were conducted comparing lipids between the control diet fed mice against lipids observed in the saturated fat enriched diet fed group. For these comparisons, Welch’s t-test was used, followed by Benjamini-Hochberg correction [44] to account for multiple comparisons.

For multivariate analysis, partial least squares regression (PLS) was used to generate a multivariate model which relates two data matrices. Using PLS, all plasma lipids are defined as predictor variables (X) and all HPF/CTX lipids as dependent variables (Y) simultaneously, in a multivariate model. The PLS model highlights the lipid species which are jointly and independently associated with the variation in the HPF/CTX. The final PLS model consists of plasma lipid species that account for the maximum variation in all the HPF/CTX. Two PLS models were generated, a Hippocampus-Plasma regression model and a Cortex-Plasma regression model. For analysis by partial least squares regression, data was log-transformed, centred and unit variance scaled. Missing values in the datasets were imputed using the NIPALS algorithm [45]. Double cross-validated PLS models were generated and assessed for statistical significance through permutation tests [46, 47].

Formalising the regression significance was accomplished through double cross validation of permutated datasets. A single permutation was conducted by randomising the relationship between the dependent variables and independent predictors and performing double cross validation on the permuted dataset. A total of 1,000 permuted datasets were analysed for each regression model.

For the same lipid species in both the plasma and brain regions, in the PLS model, the loadings are represented as two-dimensional plots. Each axis of this two-dimensional plots represents the first latent variable (LV 1) for the plasma lipids plotted against the hippocampus/cortex lipids.

Data analysis was performed using R version 5.2 [48] and the package “mixOmics” [49].

## Results

Following six months on their respective diets, the body mass of SFA fed mice was increased compared to CTRL fed mice (p<0.001, 23.4 ± 0.3 vs 32.3 ± 1.5 g; CTRL vs SFA). Mice were normoglycemic (data not shown) and otherwise healthy.

### Overview of lipid species analysed

**Fig 1:**
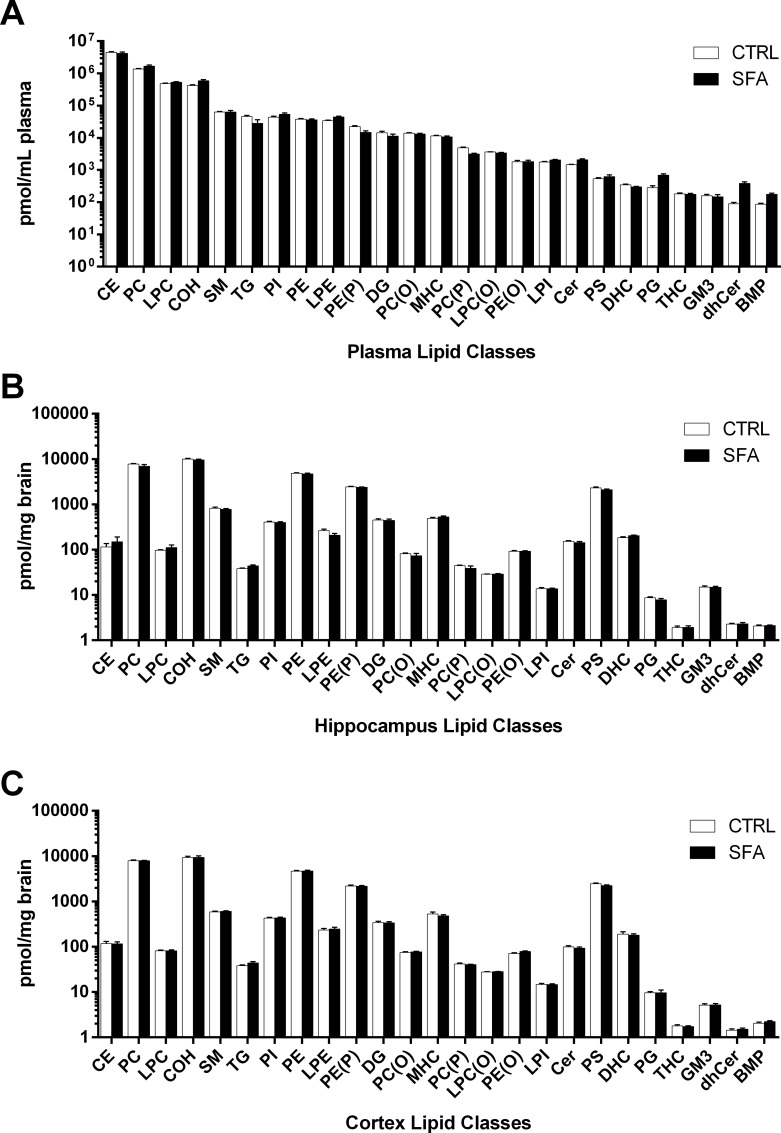
Molar abundance of lipid classes in plasma, hippocampus and cortex of mice fed regular chow or saturated fat enriched diet. Plasma, hippocampus and cortex was isolated from mice fed regular chow or an SFA enriched diet, following six months of feeding. Samples were analysed and lipids quantitated by LC-ESI-MS/MS. Molar sums were calculated by summing individual molar abundance for individual lipid species. A. Molar abundance for lipid classes in plasma in mice fed regular chow or a SFA enriched diet for six months. Order is decreasing abundance for regular chow fed animals. B. Molar abundance of lipid classes (pmol/mg wet weight) observed in the hippocampus of mice fed regular chow or an SFA enriched diet. C. Molar abundance of lipid classes (pmol/mg wet weight) observed in the cortex of mice fed regular chow or an SFA enriched diet. n = 9–10 per group. Mean ± SEM.

A total of 348 molecular lipid species were scheduled into the MRM analysis. A total of 342 were above the limit of detection and identified in plasma, 333 in the HPF and 330 in the CTX. Fig 1A depicts in decreasing order, the molar abundance for 25 lipid classes in plasma of mice maintained on normal chow or an SFA enriched diet respectively. The most abundant lipid class in plasma of mice fed a regular chow diet was cholesterol ester, comprising 63.8% of the molar abundance of lipids. The next two most abundant lipid classes were phosphatidylcholine (PC) and lysophosphatidylcholine (LPC), comprising 19.2% and 6.8% of total plasma lipids in control fed mice respectively. Unesterified cholesterol (COH) comprised 5.9% of the molar abundance of plasma lipids in control fed mice, whilst sphingomyelin (SM) > triacylglycerol (TG) > phosphatidylinositol (PI) > phosphatidylethanolamine (PE) and lysophosphatidylethanolamine (LPE) cumulatively accounted for the remaining 4.3% of plasma lipids.

A summary of the lipid ‘species’, including the most abundant species within each lipid class reported for plasma HPF and CTX is indicated in Table 2. The lipid classes indicated contained between 1–51 individual species, differing principally in acyl-chain length or degree of unsaturation. Within some lipid classes, singular lipid species made up a significant proportion of the molar abundance. For example, the fatty acyl chain 20:4 (arachidonic acid) made up 35.7% of the molar abundance of all cholesteryl ester (CE) in the plasma of mice fed regular chow. The most diverse lipid class in plasma, HPF and CTX was PC, with 51 individual species of which the most abundant (36:2) comprised 15.3% of the molar abundance of all plasma PC species.

**Table 2:** Overview of lipid classes and most abundant species identified in mice fed regular chow.

			Plasma		HPF		CTX	
Species Name	Abbreviation	No. of species	Most abundant	% of lipid class	Most abundant	% of lipid class	Most abundant	% of lipid class
Free cholesterol	COH	1	-	-	-	-	-	-
Cholesterol ester	CE	25	20:4	35.7	20:4	41.1	20:4	38.1
Diacylglycerol	DG	26	36:2	51.0	38:4	25.3	38:4	27.1
Triacylglycerol	TG	44	54:3	36.4	48:0	15.7	54:3	14.9
Bis(monoacylglycero)phosphate	BMP	1	36:2	100	36:2	100	36:2	100
Phosphatidylglycerol	PG	2	36:2	100	36:1	51.6	36:1	55.7
Phosphatidylserine	PS	5	40:6	65.8	40:6	60.7	40:6	64.4
Phosphatidylinositol	PI	16	38:4	54.9	38:4	56.2	38:4	48.3
Lysophosphatidylinositol	LPI	4	20:4	67.1	18:0	65.2	18:0	61.9
Phosphatidylethanolamine	PE	20	38:6	20.8	40:6	27.3	40:6	35.8
Alkylphosphatidylethanolamine	PE(O)	12	38:4	27.4	38:4	20.6	38:4	19.7
Alkenylphosphatidylethanolamine	PE(P)	11	40:5	41.1	38:5	23.3	40:6	21.1
Lysophosphatidylethanolamine	LPE	6	22:6	32.1	22:6	50.8	22:6	54.2
Phosphatidylcholine	PC	51	36:2	15.3	34:1	28.2	34:1	29.5
Alkylphosphatidylcholine	PC(O)	19	34:1	18.8	34:1	49.6	34:1	50.3
Alkenylphosphatidylcholine	PC(P)	13	38:5	32.1	32:0	24.1	34:3	22.5
Lysophosphatidylcholine	LPC	22	16:0	29.8	16:0	36.9	16:0	37.3
Lysoalkylphosphatidylcholine	LPC(O)	10	20:0	85.3	20:0	100	20:0	100
Sphingomyelin	SM	21	34:1	48.7	36:1	81.0	36:1	75.7
Ceramide	Cer	10	18:1	31.8	18:0	85.9	18:0	78.9
Dihydroceramide	dhCer	6	18:0	37.8	18:0	100	18:0	92.2
Monohexosylceramide	MHC	6	24:1	34.1	24:1	60.6	24:1	59.6
Dihexosylceramide	DHC	6	18:0	54.5	18:0	92.1	18:0	92.7
Trihexosylceramide	THC	6	18:0	100	18:0	100	18:0	100
GM3 ganglioside	GM3	6	24:1	52.7	18:0	84.6	18:0	82.9

**Fig 2:**
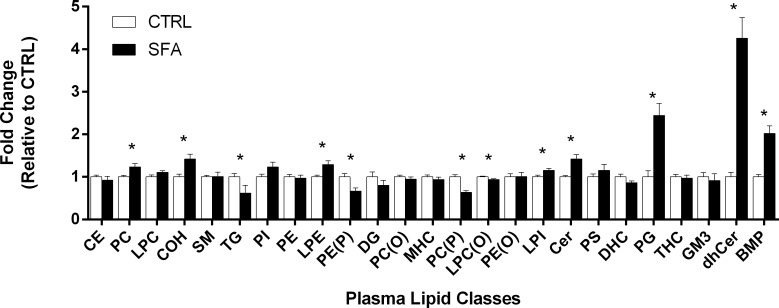
Fold changes in the molar abundance of lipid classes in plasma of mice fed regular chow or saturated fat enriched diet. Plasma was collected from mice fed a regular chow or saturated fat diet for six months. Individual lipid species were quantitated with LC-ESI-MS/MS and the molar sum across lipid classes calculated. Changes in the molar abundance of lipid classes in plasma of mice fed regular chow or saturated fat enriched diets. Fold changes in lipid classes are relative to mean lipid class concentration in mice fed regular chow diets. Lipid classes are in the order of decreasing abundance for regular chow fed animals. * indicates p<0.05, data compared using Welch’s T-test and corrected for multiple comparisons by Benjamini-Hochberg. n = 9–10 per group. Mean ± SEM.

Provision of a diet enriched in SFA for six months was well tolerated. Absolute molar abundance of plasma lipids in mice fed SFA for each lipid class are indicated in Fig 1A. The dietary induced changes in plasma lipid classes as a proportion of that found in control mice is illustrated in Fig 2. In mice fed the SFA enriched diet, CE increased 41.8%; (p = 0.005) compared to control fed mice of same age. Of the neutral lipids, plasma TG was significantly decreased in SFA fed mice compared to controls (38%; p = 0.004). Plasma diacylglycerol (DG) and CE content did not change as a consequence of long-term SFA feeding. The abundance of several plasma phospholipid classes was significantly altered as a consequence of SFA feeding. Compared to controls, SFA fed mice had a 102% increase in plasma bis(monoacylglycero)phosphate (BMP) (p<0.001); a 144% increase in phosphatidylglycerol (PG) (p<0.001) and heightened plasma PC 23.5% (p = 0.017). Of the lyso-phospholipids in plasma, in SFA fed mice there was a 15.4% increase in lysophosphatidylinositol (LPI) (p = 0.019) and a 28.9% increase in LPE (p = 0.0096). In contrast, lysoalkylphosphatidylcholine (LPC(O)) was 6.1% less in mice maintained on the SFA enriched chow diet compared to controls (p = 0.019). Significant changes in plasma alkenylphosphatidylethanolamine (PE(P)) and alkenylphosphatidylcholine (PC(P)) were identified in mice fed SFA. There was a 33.5% reduction in plasma PE(P) (p = 0.005) and PC(P) was 36.3% (p<0.001) less than control fed mice. Of the sphingolipids analysed in plasma, the SFA diet resulted in an increase in ceramide (Cer) (42.3%, p = 0.002) and a substantial increase in the precursor class, dihydroceramide (dhCer) (326%, p<0.001).

The brain lipidome in mice maintained on ordinary chow, or a diet enriched in SFA is depicted for HPF and CTX in Fig 1B and 1C, respectively. For both HPF and CTX, the brain lipidome was substantially different from that in plasma. Key qualitative differences between brain regions and plasma were proportionally greater abundance of PC, COH, PE, PE(P), monohexosylceramide (MHC), phosphatidylserine (PS) and Cer. Relative to total molar abundance in brain versus plasma; CE was 0.4% in HPF versus 63.8% plasma; PC 25.4% HPF versus 19.2% plasma, LPC 0.3% HPF versus 6.8% plasma, COH 32.5% HPF versus 5.9% plasma; SM 2.7% HPF versus 0.9% plasma, PE 15.8% HPF versus 0.5% plasma, PE(P) 7.9% HPF versus 0.3% plasma, PS 7.5% HPF versus 0.02% plasma, MHC 1.6% HPF versus 0.2% plasma and Cer 0.4% HPF versus 0.02% plasma. A similar relative abundance profile was observed for CTX samples, with relative abundance in decreasing order of COH (31.5%), PC (26.9%), PE (15.9%), PS (8.3%) and PE(P) (7.5%) of total lipids respectively.

**Fig 3:**
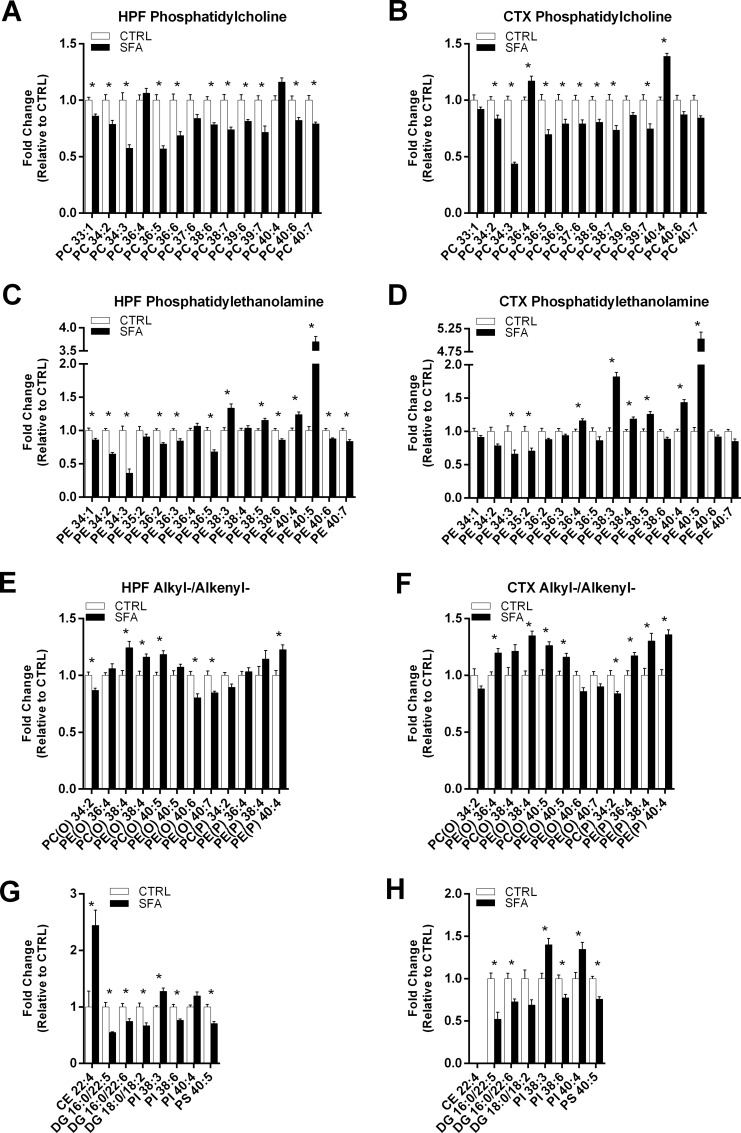
Fold change in individual lipid species in the hippocampus and cortex of mice fed a saturated fat enriched diet, relative to mice fed regular chow. Hippocampus and cortex was collected from mice fed a regular chow or saturated fat diet for six months. Individual lipid species were quantitated with LC-ESI-MS/MS. Fold changes in individual lipid species are relative to mean lipid species concentration in mice fed regular chow diets. Phosphatidylcholine species that significantly differed between mice in the (A) hippocampus and (B) cortex. Phosphatidylethanolamine species that significantly differed between mice in the (C) hippocampus and (D) cortex. Alkyl- and Alkenyl- phospholipid species that significantly differed between mice in the (E) hippocampus and (F) cortex. Other lipid species that significantly changed following consumption of a saturated fat enriched diet in the (G) hippocampus and (H) cortex. * indicates p<0.05, data compared using Welch’s T-test and corrected for multiple comparisons by Benjamini-Hochberg method. n = 9–10 per group. Mean ± SEM.

Absolute changes in the brain lipidome as a consequence of long-term SFA feeding is indicated in Fig 1B and 1C. Unlike plasma, gross substantial changes in the abundance of lipid classes was not identified for HPF lipids and indeed limited to alkylphosphatidylethanolamine (PE(O)) for CTX (increased 10%; p = 0.04). However, significant changes in particular lipid species were identified within HPF and CTX as a consequence of SFA treatment (Fig 3). A total of 50 individual lipid species significantly changed and were restricted to the lipid classes PC, PE, alkylphosphatidylcholine PC(O), PC(P), PE(O), PE(P), CE, DG, PI and PS.

Total abundance of the PC class did not change following SFA treatment, however there were 11 PC species that significantly decreased in the HPF (Fig 3A); PC species 33:1, 34:2, 34:3, 36:5, 36:6, 38:6, 38:7, 39:6, 39:7, 40:6 and 40:7. Similarly in the CTX, nine PC species decreased (34:2, 34:3, 36:5, 36:6, 37:6, 36:6, 38:7 and 39:7) while PC 40:4 increased (Fig 3B).

Of the second most abundant phospholipid class of the hippocampus, PE, species 34:1, 34:2, 34:3, 36:2, 36:3, 36:5, 38:6, 40:6 and 40:7 significantly decreased following high fat feeding, while 38:3, 38:5, 40:4 and 40:5 significantly increased (Fig 3C). Fewer species were significantly changed in the CTX; PE species 36:4, 38:3, 38:4, 38:5, 40:4 and 40:5 increased in SFA fed mice, while 34:3 and 35:2 significantly decreased (Fig 3D).

Within the HPF, a number of alkyl- and alkenyl- species changed following SFA treatment. Six alkyl- species changed in the HPF; PC(O) 34:2, PE(O) 40:6 and PE(O) 40:7 significantly decreased, while PC(O) 38:4, PC(O) 40:5 and PE(O) 38:4 significantly increased (Fig 3E). The only change in akenyl- species was an increase in PE(P) 40:4. In contrast, there were seven species that significantly increased in the CTX of SFA fed mice; PC(O) 40:5, PE(O) 36:4, PE(O) 38:4, PE(O) 40:5, PE(P) 36:4, PE(P) 38:4 and PE(P) 40:4 (Fig 3F). The alkyl-/alkenyl- species to significantly decrease in the CTX was PC(P) 34:2 (Fig 3F).

Three additional HPF phospholipids were associated with SFA feeding; PI 38:6 and PS 40:5 significantly decreased, while PE 38:3 decreased (Fig 3G). Similar changes were observed in the CTX of SFA fed mice, except with the provision that PI 40:4 also significantly increased. Four neutral lipids changed in the HPF of SFA fed mice were identified; CE 22:4 increased while three DG species decreased (16:0/22:5, 16:0/22:6 and 18:0/18:2).

Partial least squares regression analysis was performed to explore the hypothesis that the regional specific changes in HPF and CTX lipidome were associated with changes in the plasma lipidome. Both PLS regression models were statistically significant as determined by permutation tests (p<0.001). The first latent variable (LV 1) explained 46.4% of the variance in the hippocampus dataset and 46.0% of the variance in the cortex dataset. Score plots of PLS regression models (S1 Fig) and proportion of explained variance (R^2^) of individual lipids are shown in S2 and S3 Tables.

**Fig 4:**
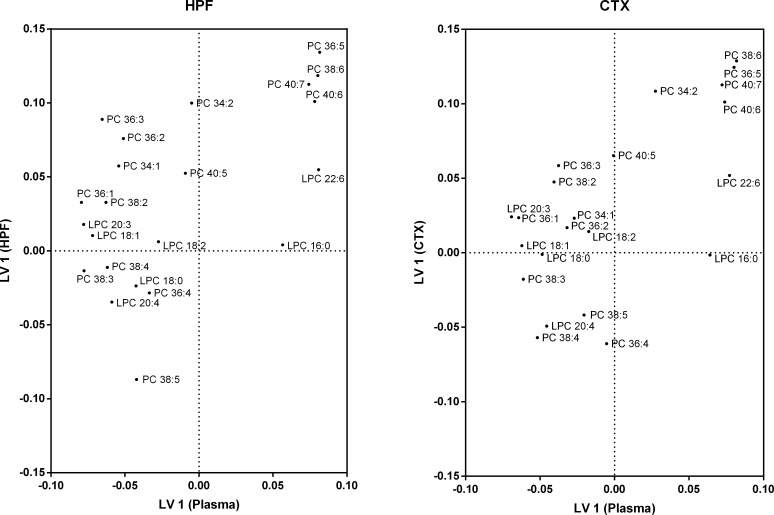
Partial least squares loading plot for phosphatidylcholine species. Wild-type mice were fed regular chow (n = 10) or a saturated fat enriched diet (n = 9) for six months. Plasma, hippocampus and cortex lipid species were quantitated with LC-ESI-MS/MS. Partial least squares regression was used to identify plasma lipid species that account for the major sources of variation within the (A) hippocampus and (B) cortex. Using the first latent variable for phosphatidylcholine species, loadings for plasma lipids are distributed on the X-axis, while loadings for hippocampus/cortex lipids are on the Y-axis. The X-axis is a measure of correlation to explaining the variation in the brain region. The Y-axis is a measure of correlation to the major source of variation in the brain region. Loadings are from the jack-knife resampled global model. Shown are the 95% most abundant PC species (22) in plasma.

Loading plots of the PLS models can be seen for PC species in Fig 4. Due to the large number of PC species identified, only the 22 most abundant plasma PC species are shown. Of these species, five (PC 36:5, PC 38:6, PC 40:7, PC 40:6 and PC 34:2) are positively correlated with the major source of variation in both hippocampus and cortex lipids. Within the plasma, PC 36:5, PC 38:6, PC 40:7 and PC 40:6 are strongly correlated with each other and also strongly correlation with the plasma species LPC 22:6. The only PC species with a strong negative correlation in the hippocampus is PC 38:5, although this was not observed in the cortex.

**Fig 5:**
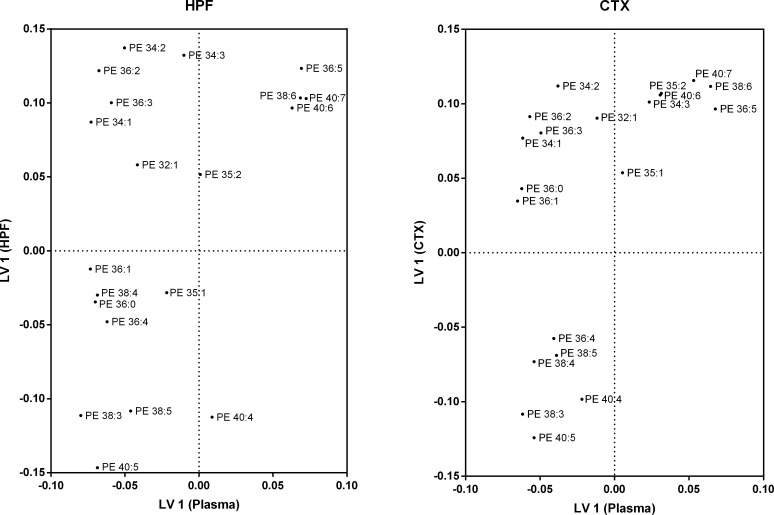
Partial least squares loading plot for phosphatidylethanolamine species. Wild-type mice were fed regular chow (n = 10) or a saturated fat enriched diet (n = 9) for six months. Plasma, hippocampus and cortex lipid species were quantitated with LC-ESI-MS/MS. Partial least squares regression was used to identify plasma lipid species that account for the major sources of variation within the (A) hippocampus and (B) cortex. Using the first latent variable for phosphatidylethanolamine species, loadings for plasma lipids are distributed on the X-axis, while loadings for hippocampus/cortex lipids are on the Y-axis. The X-axis is a measure of correlation to explaining the variation in the brain region. The Y-axis is a measure of correlation to the major source of variation in the brain region. Loadings are from the jack-knife resampled global model.

Fig 5 shows the loadings for PE species for both the HPF-Plasma and CTX-Plasma models. A group of four lipids, PE 36:5, PE 38:6, PE 40:7 and PE 40:6 correlate in both the HPF and plasma. Three lipids, PE 38:3, PE 38:5 and PE 40:5 are strongly negatively correlated to those lipids in both HPF and plasma. A number of PE species (PE 34:2, PE 36:2, PE 36:3 and PE 34:1) positively correlate with the PE 36:5, PE 38:6, PE 40:7 and PE 40:6 species in the HPF, however the plasma equivalents are positively correlated with the PE 38:3, PE 38:5 and PE 40:5 species. Similar observations are found in the CTX-Plasma model, except the strength of correlations for PE 40:6 is lower in plasma and PE 38:5 is lower in the CTX.

**Fig 6:**
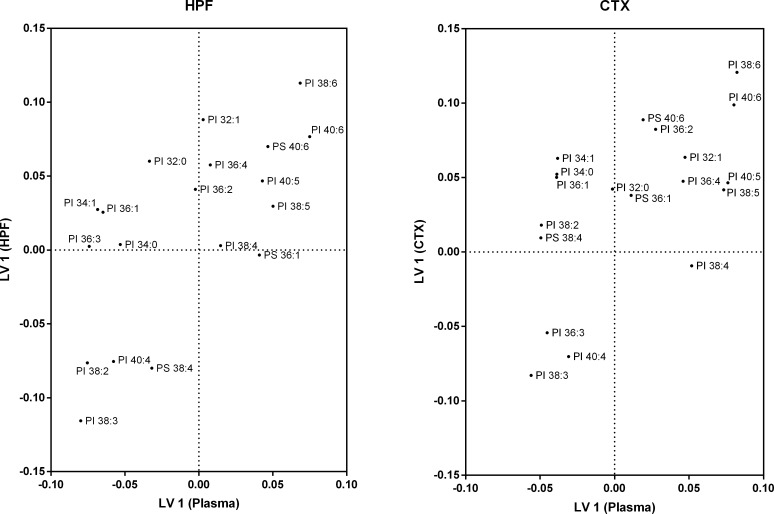
Partial least squares loading plot for phosphatidylinositol and phosphatidylserine species. Wild-type mice were fed regular chow (n = 10) or a saturated fat enriched diet (n = 9) for six months. Plasma, hippocampus and cortex lipid species were quantitated with LC-ESI-MS/MS. Partial least squares regression was used to identify plasma lipid species that account for the major sources of variation within the (A) hippocampus and (B) cortex. Using the first latent variable for phosphatidylinositol and phosphatidylserine species, loadings for plasma lipids are distributed on the X-axis, while loadings for hippocampus/cortex lipids are on the Y-axis. The X-axis is a measure of correlation to explaining the variation in the brain region. The Y-axis is a measure of correlation to the major source of variation in the brain region. Loadings are from the jack-knife resampled global model.

Loadings for the other major phospholipid classes, PS and PI, are shown in Fig 6. Two lipids (PI 38:6 and PI 38:3) contribute the most to the major source of variation in the HPF and correlate negatively with each other. Partial correlations are observed between PI 40:6 and PS 40:6 in the HPF and are partially negatively correlated with the species PI 38:2, PI 40:4 and PS 38:4. Within the CTX, PI 38:3 contributes less to variation in the cerebral lipids, while PI 40:6 contributes more. In plasma, the PI species 38:6, 40:6, 40:5 and 38:5 strongly correlate, but PI 40:5 and PI 38:5 do not correlate with other lipids that make up the major source of variation in the CTX.

## Discussion

In this study putative associations of changes in the brain lipidome with plasma lipids was determined in regular chow and saturated fat enriched diet fed mice utilizing a comprehensive lipidomics approach. Long term consumption of a SFA enriched diet realized substantial changes in the abundance of several lipid classes in plasma, including increases in PC, COH, LPE, LPI, Cer, PG, dhCer and BMP. Simultaneously, there was a decrease in the plasma abundance of TG, PE(P) and PC(P) lipid classes. In contrast, the cerebral lipidomes were relatively stable in the relative abundance of lipid classes. Hippocampus lipid classes were not different between the respective diets, while PE(O) increased slightly in CTX. Inspection of individual lipid species in the HPF/CTX revealed a total of 50 species that changed in concentration following long term consumption of the SFA enriched diet and were restricted to the lipid classes PC, PE, PC(O), PC(P), PE(O), PE(P), CE, DG, PI and PS. The regional specific changes in HPF and CTX lipidomes were assessed against associated changes in the plasma lipidome with partial least squares regression. Strong associations between a number of plasma lipids and changes in HPF and CTX lipidomes was observed. Our experiments demonstrate that well tolerated diets are capable of regulating the HPF and CTX lipidomes, while plasma lipids may be a causal mechanism for these changes. This paper suggests dietary lipids are capable of influencing cerebral lipid homeostasis, which may explain previously observed associations of dietary patterns with onset and progression of neurodegenerative disorders [50, 51].

The brain is highly enriched in lipids, where they influence neurotransmission, synaptic plasticity and inflammation. Changes in cerebral lipid homeostasis has been widely reported in a number of neurodegenerative and behavioural disorders, however, it is not known whether these changes are causally related with the onset or progression of such disorders or consequential to the disease pathology. Knowledge of factors that regulate the cerebral lipidome in a physiological context is still in its infancy. Further, animal models are routinely used to study the impact of western diets on neurological function/disorders, yet little is known about changes in the plasma and the cerebral lipidome for well tolerated diets. Herein, we explore in detail the influence a diet enriched in saturated fat has on the lipidome of the hippocampus and cerebral cortex, and the associated changes in plasma.

Following long term consumption of an SFA enriched diet, we observed an increase in the abundance of plasma PC lipids. It is not known whether bulk changes in plasma PC abundance has any effect on cerebral homeostasis. Individual PC species, on the other hand, have been noted to be associated with several neurodegenerative disorders [52, 53]. The bioactive PC isoform hypothesis may be because, next to cholesterol esters, PC lipids contain the largest plasma proportion of circulating DHA, eicosapentaenoic acid (EPA) and arachidonic acid (AA) (data not shown)–fatty acids reported to influence risk of cognitive decline. Lysophosphatidylcholine, a product of enzyme hydrolysis of PC lipids, has been proposed to be a major route for peripheral uptake by the brain [54, 55], although the plasma abundance was not altered in these mice.

Plasma non-esterified cholesterol has long been known to be associated with many conditions, including coronary heart disease, Alzheimer’s disease and vascular mortality [56]. Elevated plasma COH has previously been reported to be associated with alternations in cerebral lipid homeostasis [57]. Stranahan et al. speculated this was through an oxidative mechanism, however causal associations with plasma lipids was not assessed. A surprising result observed in this study was the decrease in plasma TG. Plasma TG concentrations are predominantly determined by the rate of production/secretion of triglyceride-rich lipoproteins and by the rate of hydrolysis/removal of circulating TG. Given our SFA enriched diet contains approximately 5-fold more dietary fat, we might expect to observe an increase in circulating TG. The contribution of circulating TG to brain lipid uptake is unclear, as a majority of research on brain uptake of peripheral lipids have focused on non-esterified fatty acids and LPC [55]. However, further research will be needed to clarify this difference, as triacylglycerol-rich lipoproteins and their lipolysis products in high physiological concentrations are known to cause endothelial injury and dysfunction in the periphery and in the cerebral circulation [33].

Sphingolipids have long been known to be responsive to SFA enriched diets and thought to be responsible for many of the deleterious effects, including insulin resistance, oxidative stress and inflammation [58, 59]. Herein, we observed increases in the plasma concentration of ceramides and its precursor dihydro-ceramides. Several cohort studies have identified circulating sphingolipids as potential biomarkers of neurodegenerative diseases [60–62]. Lyn-Cook et al. proposed that circulating ceramides might directly enter the brain, due to their hydrophobicity [63]. However, in this study, there was no observable change in cerebral sphingolipids.

An interesting finding in this study was a considerable decrease in the concentration of alkenylphosphatidylethanolamine and alkenylphosphatidylcholine classes in plasma following long term consumption of an SFA enriched diet. These lipid classes have been proposed to have a protective role against oxidative stress and lower concentrations associated with cardiovascular and neurodegenerative disorders [64, 65]. To the authors knowledge, this study is the first report of decreases in plasma PE(P) and PC(P) in mice fed a saturated fat enriched diet. This result is particularly interesting because serum PE(P) was noted to be able to predict severity of dementia in humans [66]. Further, serum PE(P) concentrations was shown to decrease prior to the detectable symptoms of dementia, suggesting changes in plasma ether lipids may have an early involvement in neurodegenerative disease progression.

Changes in the cerebral lipidome to dietary interventions have principally focused on n-3 fatty acid modulation and in many situations represent very severe restrictions [67]. The cerebral lipidome modulating ability of high-(saturated) fat feeding is considerably less well known. In our study, we report a significant increase in the abundance of PE(O) lipid species in the cerebral cortex of mice fed a modest saturated fat enriched diet. As PE(P) was not changed and PE(O) is a direct biosynthetic precursor to PE(P), this mismatch may be indicative of early perturbations in ether-lipid synthesis and/or peroxisomal stress [68]. The saturated ether linkage in PE(O) is not particularly reactive to oxidative stress, unlike the vinyl-ether linkage in PE(P), so increased PE(O) may be indicative of an increased flux through the pathway to replace PE(P) lost to reactive oxygen species (ROS) scavenging. To further test this hypothesis, studies examining the oxidative products of PE(P) species could be performed.

Recent findings also confirm that high-fat feeding can influence different regions of the brain. Borg et al. fed C57Bl/6 mice a 60% energy from fat diet for 8 weeks and observed increases in DG, TG, PC(O), dhCer, DHC and BMP lipid classes in the hypothalamus [11]. Further, they note increases in a number of individual Cer species. The hypothalamic lipid accumulation in response to the high-fat diet was not ameliorated by exercise, which has been shown to reduce lipid accumulation in many peripheral tissues. The accumulation of TG, dhCer and Cer may indicate that the supply of lipids is surpassing the ability of neuronal cells to effectively handle the quantity of fatty acids. This observation is supported by Posey et al. whom observed an increase in saturated long chain acyl-CoA species in the hypothalamus of rats fed a similar diet i.e. accumulation of early lipid metabolism intermediates [13]. These studies examined the hypothalamus, which has roles in modulating energy intake through fatty acid sensing neurons within this region. This may predispose this region to being more susceptible to lipid accumulation. However, a study by Stranahan et al. using an aggressive high-fat diet in rats, showed an accumulation in the hippocampus of non-esterified cholesterol, galactosyl ceramide, ceramide sulfatide and several isoforms of sphingomyelin [57]. It should be noted that only hippocampus tissue of animals whose serum cholesterol levels fell into the highest third where selected; limiting generalizability of results. Nonetheless, there is a growing number of high-fat feeding studies which indicate sphingolipid species to be elevated in various brain regions.

In addition to the change in lipid class abundance, we observed a large number of changes in individual lipid species in both the hippocampus and cortex. Significant changes were observed in many phospholipid classes, as well as the DG class and one species changed in the cholesterol esters. A possible explanation for the large number of changes in phospholipid classes is due to their continuous turnover. Neuronal membrane phospholipids are repeatedly hydrolysed by phospholipase A1/2 enzymes and re-esterified—a cyclical process which is crucial for endo/exi-cytosis of vesicles during neurotransmitter release and membrane fusion events [69]. The deacylation/reacylation provides opportunity for phospholipid remodelling, where the local activity of enzymes and fatty acid concentrations interact and determine phospholipid composition [55]. We observed similar changes in both the hippocampus and cortex of mice fed the saturated fat enriched diet, suggesting a high degree of co-regulation between the regions. Considering the changes were broadly reflected in both regions, it is possible that peripheral supply of fatty acids is major factor in phospholipid composition.

Few studies have described in detail the changes in individual lipid species within the brain in high-fat feeding studies. However, some evidence suggests robust changes in phospholipid composition in response to high-fat feeding. Yu et al. performed a study in mice with multi-generation feeding with a high-lard diet and reported cerebral fatty acid composition using gas-chromatography [70]. Compared to control-diet fed mice, the high-lard diet group exhibited a fatty acid profile that contained significantly less polyunsaturated fatty acids and significantly more saturated fatty acids. This coincided with a halving in DHA content in the brain. Lepinay et al. fed pregnant wistar rats and their pups a high-fat diet through to adulthood and measured the fatty acid composition of PE lipids in the hippocampus [71]. The high-fat diet resulted in a significant reduction in n-3 fatty acids and an increase in n-6 fatty acids, while total polyunsaturated fatty acids did not change. This implicates a replacement of n-3 fatty acids with n-6 fatty acids is occurring within the PE class of the hippocampus.

Examination of the individual species in the brains of our mice which significantly differed, reveals that many of the lipids that putatively contain DHA decreased, and those containing AA increased. The replacement of DHA by AA has been reported previously in studies of severe n3-fatty acid deficiency, however these changes were principally only observed in PC and PE lipids [67]. By contrast, a recent study by Bascoul-Colombo et al. assessed cerebral lipids following long-term provision of a diet with 10-fold greater n3-fatty acids by way of DHA supplementation and reported a replacement of AA containing phospholipids by DHA in the hippocampus, cortex and cerebellum [72]. While the relative composition of the different regions varied, the changes in individual lipids all trended in the same direction among the phospholipid classes assessed. This suggests that dietary lipids can influence the composition of many phospholipid classes simultaneously.

The most novel component of this study is the simultaneous multivariate analysis of whole lipidomes from both plasma and brain. Whereas there has been attempts to correlate cerebral lipids with circulating lipids, these have been limited to cerebral fatty acid profiles against measures of serum cholesterol [70]. By contrast, we explore relationships between all plasma lipids and all cerebral lipids observed in our detailed lipidomics study. This approach is particularly relevant for lipidomic studies as the complex relationships within and between different compartments (plasma vs brain) can be examined. Furthermore, changes in individual lipids in the brain are generally small because the influencing plasma lipids are metabolised and distributed among many lipids.

Partial least squares regression used in this study shows that DHA and AA containing phospholipids contribute, opposingly, to a major source of variation observed in our dataset. The plasma lipidome, as a whole, was able to account for a substantial proportion of variation in the brain, strengthening the suggestion that plasma lipids are causally related to lipidome changes in the brain.

A number of assumptions were made during this study: (1) the number of internal standards are limited compared to the number of species measured. It is assumed that one internal standard is representative of the entire class, with minimal differences in response factor with changes in fatty acid composition; (2) there may be some degradation of lipids during sample isolation and processing. However, the hippocampus and cortex were isolated from brain sections that were snap frozen immediately after collection; (3) the same mass spectrometry analysis of lipids was performed on both plasma and brain samples. While some studies have detailed novel and specific species in plasma [73] and brain [74], we have attempted to focus on the major species present in these samples. Further, the number of species identified, 348, provides an ample number to characterise a vast number of the major species present in the samples; (4) we did not perfuse the brain prior to sample collection. Plasma lipids will contribute to the cerebral lipids measured, however their relative abundance will be negligible given the volume of plasma within the brain compared to tissue mass. As evidenced by the differential composition of plasmalogens and cholesterol esters between plasma and brain regions. (5) Internal validation does not preclude the need for external validation of the PLS models generated. Further research is required to externally validate the findings [75]. Despite these potential limitations, the relative changes between groups are still accurate as each animal was processed the same. Furthermore, by utilizing multivariate analysis of plasma with the two brain regions from each animal allows correlations to be observed and biological phenomenon to be characterised independent of intra-sample systematic error [76].

Herein, we show that long-term feeding of SFA enriched diets in mice leads to changes in the hippocampus and cerebral cortex lipidomes. Similar patterns of change are observed between the two regions of the brain, suggesting a common causal mechanism. Whole lipidome multivariate analysis suggests the plasma lipidome can account for a substantial proportion of variation in both the hippocampus and cortex. Given that well tolerated diets can alter cerebral lipid composition, future clinical work should consider ‘whole diets’, rather than single dietary components i.e. DHA.

## Supporting Information

S1 Fig Scores scatter plot for partial least squares models. Wild-type mice were fed regular chow (n = 10) or a saturated fat enriched diet (n = 9) for six months. Plasma, hippocampus and cortex lipid species were quantitated with LC-ESI-MS/MS. Partial least squares regression was used to identify plasma lipid species that account for the major sources of variation within the (A) hippocampus and (B) cortex. Scores scatter plot of the first latent variable for mice fed regular chow (squares) or a saturated fat enriched diet (triangles). The first latent variable score for the independent lipids (plasma) on the X-axis and the first latent variable score for dependent lipids (hippocampus/cortex) on the Y-axis. Scores are from the jack-knife resampled global model.
(TIF)

S1 Table Conditions for tandem mass spectrometry analysis of lipid species (DOCX)

S2 Table Proportion of variance explained (R^2^) by the first latent variable for the first 25 individual lipids in the hippocampus and plasma lipids. (DOCX)

S3 Table Proportion of variance explained (R^2^) by the first latent variable for the first 25 individual lipids in the cerebral cortex and plasma lipids. (DOCX)
