# Supplementary figures and images for: The Effects of Long-Term Saturated Fat Enriched Diets on the Brain Lipidome
Source: PLoS One. 2016 Dec 1;11(12):e0166964. doi: 10.1371/journal.pone.0166964 (PMC5132325; doi:10.1371/journal.pone.0166964)

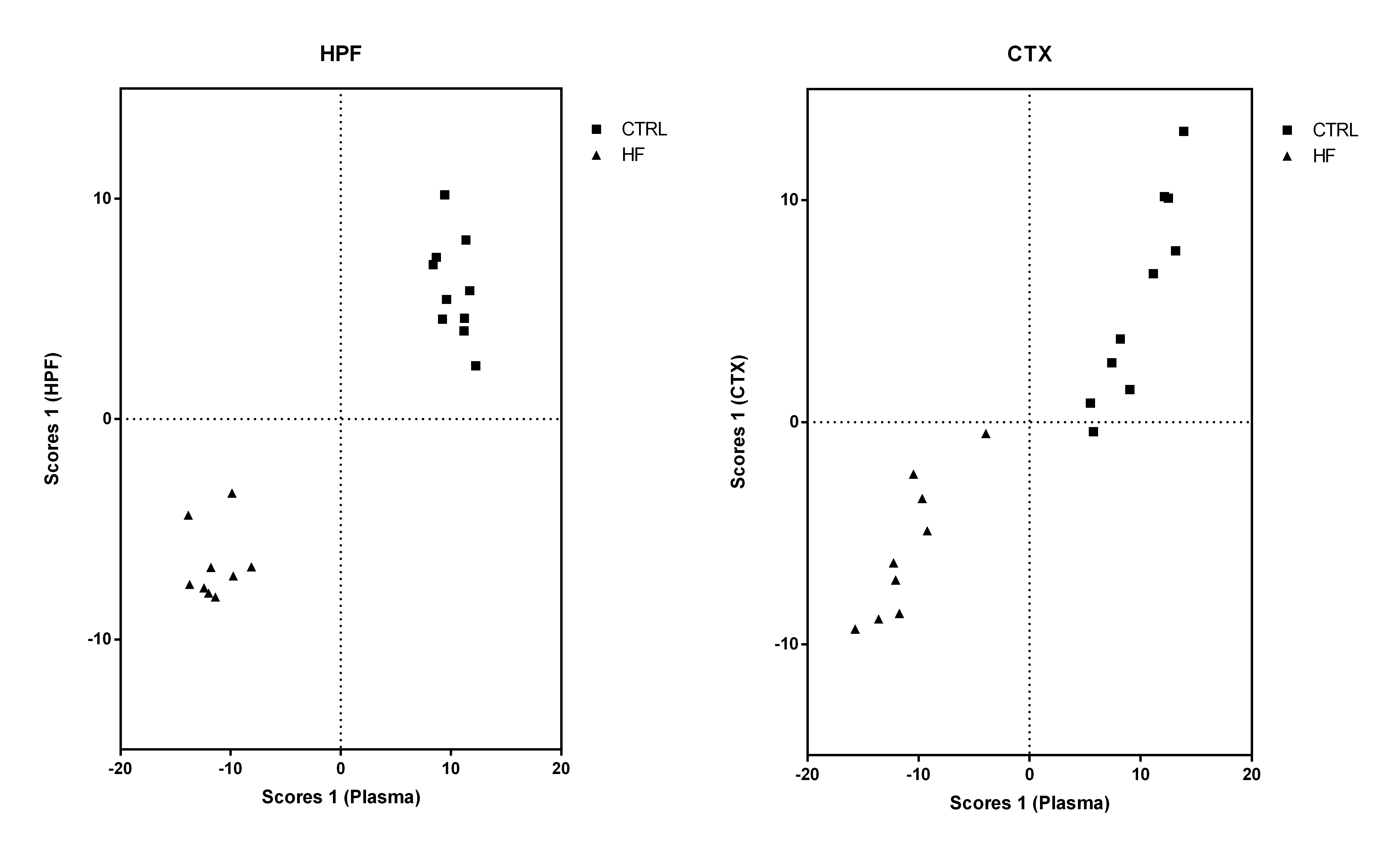

Supplement: S1 Fig — Wild-type mice were fed regular chow (n = 10) or a saturated fat enriched diet (n = 9) for six months. Plasma, hippocampus and cortex lipid species were quantitated with LC-ESI-MS/MS. Partial least squares regression was used to identify plasma lipid species that account for the major sources of variation within the (A) hippocampus and (B) cortex. Scores scatter plot of the first latent variable for mice fed regular chow (squares) or a saturated fat enriched diet (triangles). The first latent variable score for the independent lipids (plasma) on the X-axis and the first latent variable score for dependent lipids (hippocampus/cortex) on the Y-axis. Scores are from the jack-knife resampled global model. (TIF) [file pone.0166964.s001.tif]
