# Supplementary material for: The Effects of Long-Term Saturated Fat Enriched Diets on the Brain Lipidome
Source: PLoS One. 2016 Dec 1;11(12):e0166964. doi: 10.1371/journal.pone.0166964 (PMC5132325; doi:10.1371/journal.pone.0166964)
Supplement: S1 Table — (DOCX) [file pone.0166964.s002.docx]

**S1 Table:** **Conditions for tandem mass spectrometry analysis of lipid species**

| **Lipid class or subclass** | **No. of species** | **Internal standard** | **Pmol^a^** | **Parent ion** | **Experiment^b^** | **Voltage settings^c^** | | | |
| --- | --- | --- | --- | --- | --- | --- | --- | --- | --- |
|  |  |  |  |  |  | **DP** | **EP** | **CollE** | **CXP** |
| Dihydroceramide (dhCer) | 6 | dhCer 8:0 | 100 | [M+H]^+^ | PIS, 284.3 *m/z* | 90 | 30 | 28 | 10 |
| Ceramide (Cer) | 10 | Cer 17:0 | 100 | [M+H]^+^ | PIS, 264.3 *m/z* | 50 | 10 | 35 | 12 |
| Monohexocylceramide (MHC) | 6 | MHC 16:0 *d_3_* | 50 | [M+H]^+^ | PIS, 264.3 *m/z* | 77 | 10 | 50 | 12 |
| Dihexosylceramide (DHC) | 6 | DHC 16:0 *d_3_* | 50 | [M+H]^+^ | PIS, 264.3 *m/z* | 100 | 10 | 65 | 12 |
| Trihexosylceramide (THC) | 6 | THC 17:0 | 50 | [M+H]^+^ | PIS, 264.3 *m/z* | 130 | 10 | 73 | 12 |
| G_M3_ ganglioside (GM3) | 6 | THC 17:0 | 50 | [M+H]^+^ | PIS, 264.3 *m/z* | 155 | 10 | 105 | 16 |
| Sphingomyelin (SM) | 21 | SM 12:0 | 200 | [M+H]^+^ | PIS, 184.1 *m/z* | 65 | 10 | 35 | 12 |
| Phosphatidylcholine (PC) | 51 | PC 13:0/13:0 | 100 | [M+H]^+^ | PIS, 184.1 *m/z* | 100 | 10 | 45 | 11 |
| Alkylphosphatidylcholine (PC(O)) | 19 | PC 13:0/13:0 | 100 | [M+H]^+^ | PIS, 184.1 *m/z* | 100 | 10 | 45 | 11 |
| Alkenylphosphatidylcholine (PC(P)) | 13 | PC 13:0/13:0 | 100 | [M+H]^+^ | PIS, 184.1 *m/z* | 100 | 10 | 45 | 11 |
| Lysophosphatidylcholine (LPC) | 22 | LPC 13:0 | 100 | [M+H]^+^ | PIS, 184.1 *m/z* | 90 | 10 | 38 | 12 |
| Lysoalkylphosphatidylcholine (LPC(O)) | 10 | LPC 13:0 | 100 | [M+H]^+^ | PIS, 285.2 *m/z* | 90 | 10 | 42 | 5 |
| Phosphatidylethanolamine (PE) | 20 | PE 17:0/17:0 | 100 | [M+H]^+^ | NL, 141 Da | 80 | 10 | 31 | 7 |
| Alkylphosphatidylethanolamine (PE(O)) | 12 | PE 17:0/17:0 | 100 | [M+H]^+^ | NL, 141 Da | 80 | 10 | 31 | 7 |
| Alkenylphosphatidylethanolamine (PE(P)) | 11 | PE 17:0/17:0 | 100 | [M+H]^+^ | NL, 141 Da | 80 | 10 | 31 | 7 |
| Lysophosphatidylethanolamine (LPE) | 6 | PE 14:0/0:0 | 100 | [M+H]^+^ | NL, 141 Da | 80 | 10 | 31 | 7 |
| Phosphatidylinositol (PI) | 16 | PE 17:0/17:0 | 100 | [M+NH_4_]^+^ | NL, 277 *m/z* | 51 | 10 | 43 | 14 |
| Lysophosphatidylinositol (LPI) | 4 | PE 14:0/0:0 | 100 | [M+NH_4_]^+^ | NL, 277 *m/z* | 51 | 10 | 43 | 14 |
| Phosphatidylserine (PS) | 5 | PS 17:0/17:0 | 100 | [M+H]^+^ | NL, 185 Da | 86 | 10 | 29 | 16 |
| Phosphatidylglycerol (PG) | 2 | PG 17:0/17:0 | 100 | [M+NH_4_]^+^ | NL, 189 Da | 60 | 10 | 25 | 12 |
| Cholesterol ester (CE) | 25 | CE 18:0 *d_6_* | 1000 | [M+NH_4_]^+^ | PIS, 369.3 *m/z* | 30 | 10 | 20 | 12 |
| Free cholesterol (COH) | 1 | COH *d_7_* | 1000 | [M+NH_4_]^+^ | PIS, 369.3 *m/z* | 55 | 10 | 17 | 12 |
| Diacylglycerol (DG) | 26 | DG 15:0/15:0 | 200 | [M+NH_4_]^+^ | NL, fatty acid | 55 | 10 | 30 | 22 |
| Triacylglycerol (TG) | 44 | TG 17:0/17:0/17:0 | 100 | [M+NH_4_]^+^ | NL, fatty acid | 95 | 10 | 30 | 12 |
| Bis(monoacylglycero)phosphate (BMP) | 1 | BMP 14:0/14:0 | 100 | [M+NH_4_]^+^ | NL, fatty acid dependent | 65 | 10 | 35 | 5 |

^a^ Amount of internal standard per sample

^b^ PIS = precursor ion scan, NL = neutral loss scan.

^c^ DP = declustering potential (volts); EP = entrance potential (volts); CollE = collision energy (volts); CXP = collision cell exit potential (volts).
