# Supplementary material for: The Effects of Long-Term Saturated Fat Enriched Diets on the Brain Lipidome
Source: PLoS One. 2016 Dec 1;11(12):e0166964. doi: 10.1371/journal.pone.0166964 (PMC5132325; doi:10.1371/journal.pone.0166964)
Supplement: S3 Table — (DOCX) [file pone.0166964.s004.docx]

**S3 Table. Proportion of variance explained (R^2^) by the first latent variable for the first 25 individual lipids in the cerebral cortex and plasma lipids.**

| Cortex | | Plasma | |
| --- | --- | --- | --- |
| **Lipid** | **R^2^** | **Lipid** | **R^2^** |
| PC 38:6 | 0.87 | PC 39:6 | 0.96 |
| PE 34:2 | 0.85 | PC 36:5 | 0.96 |
| PC 40:7 | 0.85 | PC 38:7 | 0.96 |
| PC 38:7 | 0.80 | CE 20:5 | 0.96 |
| PC 28:0 | 0.80 | PC 36:6 | 0.96 |
| PE 40:7 | 0.79 | PC 37:6 | 0.95 |
| PI 38:6 | 0.79 | PC(P) 38:6 | 0.94 |
| PC 36:6 | 0.76 | PC 31:0 | 0.94 |
| PE(P) 38:6 | 0.73 | TG 16:0/18:1/18:2 | 0.93 |
| PC 40:6 | 0.73 | PC(P) 38:5 | 0.93 |
| PC 37:6 | 0.72 | LPC 22:6 | 0.93 |
| PS 40:5 | 0.71 | PC(O) 40:7 | 0.92 |
| PE(P) 40:6 | 0.70 | CE 14:0 | 0.92 |
| PC(P) 38:6 | 0.70 | PC 38:6 | 0.92 |
| PE(O) 40:6 | 0.70 | PC 34:5 | 0.92 |
| PE 40:6 | 0.69 | TG 16:1/18:1/18:2 | 0.91 |
| PE 38:6 | 0.69 | PC(P) 36:5 | 0.91 |
| PC 34:3 | 0.67 | PI 40:6 | 0.91 |
| PC 36:5 | 0.65 | TG 16:0/18:2/18:2 | 0.91 |
| PC(P) 34:2 | 0.65 | TG 18:1/18:1/18:2 | 0.90 |
| PI 40:6 | 0.65 | TG 18:1/18:1/22:6 | 0.90 |
| PC 39:6 | 0.64 | PE(O) 40:5 | 0.89 |
| PC 39:7 | 0.63 | LPC 20:5 | 0.89 |
| PC 34:2 | 0.62 | LPC 22:5 | 0.88 |
| PC 35:1 | 0.62 | TG 16:0/16:1/18:1 | 0.88 |
